# Collective information storage in multiple synapses enables fast learning and slow forgetting

**DOI:** 10.1186/1471-2202-16-S1-O15

**Published:** 2015-12-18

**Authors:** Michael J Fauth, Florentin Wörgötter, Christian Tetzlaff

**Affiliations:** 1 Bernstein Center for Computaional Neuroscience, Third Physics Institute, Göttingen, Germany; 2 Max Planck Institute for Dynamics and Self-Organization, Göttingen, Germany; 3 Weizman Institute, Rehovot, Israel

## 

Most of the excitatory cortical synapses reside on dendritic spines. Although these spines undergo a remarkably high turnover [1,2], they have been shown to be involved in learning and long-term memory. Along this line, it is unclear how information is preserved while its substrate (synapses or spines) is permanently changing.

Here, we use a simple stochastic model of structural plasticity to investigate this phenomenon : We assume a certain number of potential synaptic locations from one neuron to another.

At those locations, synapses (spines) are created with a constant probability and removed with a probability depending on the number of existing synapses and the stimulation of the neurons. From these two probabilities, the stationary distribution of the number of synapses between two neurons can be calculated.

Experimental measurements of these stationary probability distributions in the cortex show that the majority of connections has either zero or multiple synapses while one or two contacts are very improbable [e.g., [3-5]]. Using information theoretic measures we show that, in our model, such bimodal distributions enable information storage over time scales many orders of magnitudes higher than the involved probabilities. Thus, in this system the conflict of rapid spine turnover (probabilities) and long-term memory is resolved by storing the information collaboratively in multiple synapses.

In the following, we will consider the bimodal stationary distributions as the working point of the system. Then, we can model external signals, as, e.g., increased or decreased activities during learning, as changes of the removal probabilities and stationary distributions (e.g., mediated by synaptic plasticity [6]).

For instance, for learning signals resulting to unimodal stationary distributions (only connected or only unconnected), we find that learning is orders of magnitude faster than forgetting. Along this line, we observe that retraining a task does not induce an increased overturn rate as during initial training, which has been similarly observed for dendritic spines in vivo [1,2]. Our results clearly relate the difference in time scales to the shape of the stationary distribution and therefore reveal the functional advantage of the bimodal distribution found in experiment.

## References

[B1] Yang G Pan F Gan WB Stably maintained dendritic spines are associated with lifelong memories Nature 2009 462 920 924 19946265 10.1038/nature08577 PMC4724802

[B2] Xu T Yu X Perlik AJ Tobin WF Zweig JA Tennant K Rapid formation and selective stabilization of synapses for enduring motor memories Nature 2009 462 915 919 19946267 10.1038/nature08389 PMC2844762

[B3] Feldmeyer D Egger V Lübke J Sakmann B Reliable synaptic connections between pairs of excitatory layer 4 neurones within a single 'barrel' of developing rat somatosensory cortex J Physiol 1999 521 1 169 190 10562343 10.1111/j.1469-7793.1999.00169.x PMC2269646

[B4] Feldmeyer D Lübke J Silver RA Sakmann B Synaptic connections between layer 4 spiny neurone-layer 2/3 pyramidal cell pairs in juvenile rat barrel cortex: physiology and anatomy of interlaminar signalling within a cortical column J Physiol 2002 538 Pt 3 803 822 11826166 10.1113/jphysiol.2001.012959 PMC2290091

[B5] Hardingham NR Hardingham GE Fox KD Jack JJB Presynaptic efficacy directs normalization of synaptic strength in layer 2/3 rat neocortex after paired activity J Neurophysiol 2007 97 4 2965 2975 17267749 10.1152/jn.01352.2006

[B6] Fauth M Wörgötter F Tetzlaff C The formation of multi-synaptic connections by the interaction of synaptic and structural plasticity and their functional consequences PLOS Comput Biol 2015 11 1 e1004031 25590330 10.1371/journal.pcbi.1004031 PMC4295841

